# Latent profiles and associated factors of workplace violence among nurses in tertiary hospitals: a cross-sectional study

**DOI:** 10.3389/fpsyt.2025.1651237

**Published:** 2025-09-11

**Authors:** Sumei Zhou, Zhi Zeng

**Affiliations:** ^1^ Department of Neurosurgery, Deyang People’s Hospital, Deyang, Sichuan, China; ^2^ Department of Gastroenterology, Deyang People’s Hospital, Deyang, Sichuan, China

**Keywords:** nurses, workplace violence, latent profile analysis, influencing factors, cross-sectional survey

## Abstract

**Background:**

Workplace violence (WPV) is a significant occupational hazard that threatens nurses’ psychological well-being and professional stability. Although prior studies have addressed the impact of WPV on nurses, the latent heterogeneity of their violence exposure patterns has not been systematically explored. Moreover, empirical evidence regarding the role of individual psychological traits in shaping different WPV experiences remains limited.

**Objective:**

This study aimed to identify latent profiles of WPV exposure among nurses and examine the associations between profile membership and demographic as well as psychological factors, in order to uncover key predictors of distinct WPV patterns.

**Methods:**

A cross-sectional survey was conducted between March and May 2025 among 549 registered nurses from eight tertiary hospitals in Sichuan Province, China. Participants completed a battery of standardized instruments, including General Demographic Data Scale, Workplace violence Scale, Maslach Burnout Inventory, Connor-Davidson Resilience Scale, Emotional Labor Scale, and Perceived Organizational Support Scale. Latent Profile Analysis (LPA) was performed using Mplus 8.3 to identify WPV exposure subgroups, and multivariate logistic regression was used to determine associated factors.

**Results:**

LPA revealed two distinct WPV profiles: a high-frequency, multi-type violence group (n = 152, 27.7%) and a low-frequency, mild violence group (n = 397, 72.3%). Nurses in the high-frequency group reported significantly higher scores across all WPV dimensions, including verbal abuse, sexual harassment, threats, and physical assault (*P* < 0.001). Logistic regression analysis indicated that having a bachelor’s degree or higher, lower salary satisfaction, and lower psychological resilience were significant predictors of membership in the high-frequency WPV group (*P* < 0.01).

**Conclusion:**

Nurses’ WPV experiences exhibit distinct latent profiles. Educational level, salary satisfaction, and psychological resilience are key differentiating variables. These findings highlight the need for stratified risk identification and targeted interventions, particularly for nurses with higher education levels, low salary satisfaction, and reduced psychological resilience, in order to mitigate the adverse effects of WPV and enhance occupational adaptation.

## 1 Introduction

The “14th Five-Year Plan for the Development of Nursing Services” in China emphasizes improving the quality of nursing care and strengthening the nursing workforce as essential components of the national “Healthy China” strategy (1). As a vital part of the healthcare system, clinical nurses are frequently exposed to high-intensity and high-risk working environments. Workplace violence (WPV) has emerged as a critical occupational hazard, significantly threatening nurses’ psychological well-being and professional stability (2). Evidence suggests that WPV not only undermines job satisfaction and service efficiency but also poses indirect risks to patient safety and the stability of healthcare systems (3, 4).

Surveys indicate that approximately 70% of nurses experience some form of workplace violence—ranging from verbal abuse and threats to physical assault—during their careers (5). These exposures are associated with increased risks of anxiety, depression, and burnout, and in severe cases, contribute to workforce attrition and organizational disengagement (6, 7). Accordingly, identifying WPV risk profiles and clarifying its psychological impact mechanisms has become a priority in nursing management and policy development.

Most prior research has assessed WPV using total or average scores, which may reflect overall prevalence but fail to capture latent heterogeneity in exposure patterns across nurse subgroups (8, 9). Moreover, limited attention has been paid to the interaction between individual psychological traits and external support systems, hindering the development of targeted and effective interventions (10). Latent Profile Analysis (LPA), a person-centered statistical method, enables the identification of unobserved subgroups based on multiple WPV dimensions, and reveals differences in psychological responses and organizational adaptation across profiles, offering strong theoretical and practical value (11, 12).

In this context, the present study employed a cross-sectional design among clinical nurses in tertiary hospitals, using LPA to identify latent profiles of WPV exposure. Furthermore, the study incorporated measures of occupational burnout, emotional labor, psychological resilience, and perceived organizational support to examine psychological characteristics and underlying mechanisms across different WPV subgroups, with the aim of informing precision identification and stratified intervention strategies to improve occupational health and nursing service delivery.

## 2 Methods

### 2.1 Participants

A cross-sectional design was employed for this study. Between March and May 2025, clinical nurses were recruited from eight tertiary grade-A hospitals in Sichuan Province using convenience sampling. Inclusion criteria were as follows: (1) age ≥18 years with a valid nursing license; (2) ≥1 year of clinical work experience; and (3) informed consent and voluntary participation. Exclusion criteria included: (1) nurses in internship, advanced training, or administrative roles; (2) those on long-term sick leave or maternity leave; and (3) individuals experiencing major psychological stress during the survey period. This study was approved by the institutional ethics committee (Approval No. 2023-04-083-K01).

### 2.2 Sample size

Based on conventional sample size estimation, a minimum of 5–10 times the number of independent variables is generally recommended for multivariate analysis (13). This study included 20 key variables, indicating a required sample size between 100 and 200 participants. To enhance statistical power and model stability, and considering an expected 20% rate of invalid responses, the final estimated sample size ranged from 125 to 250.

A total of 600 questionnaires were distributed, and 549 valid responses were obtained after excluding 37 questionnaires with patterned responses or logical inconsistencies, yielding a response rate of 91.5%.

### 2.3 Data collection procedure

Online data collection was conducted using the “Questionnaire Star” platform. The nursing departments and head nurses at the participating hospitals facilitated the survey process. Trained coordinators were responsible for questionnaire distribution and respondent guidance. Nurses completed the electronic questionnaire voluntarily during work breaks after being fully informed about the study purpose. The survey strictly adhered to principles of informed consent and anonymity.

To ensure data quality, several control measures were implemented. First, required-response settings and logical skip patterns were embedded in the questionnaire to minimize missing data and contradictory answers. Second, a minimum completion time of five minutes was enforced, and questionnaires with abnormally short durations were automatically flagged for exclusion. IP and device ID tracking were used to prevent duplicate submissions. All submitted questionnaires were manually reviewed, and those with highly patterned responses, logical errors, or duplicated content were excluded.

### 2.4 Instruments

1. Demographic Information: Basic demographic and occupational characteristics were collected through a self-administered questionnaire, including gender, age, marital status, educational level, employment type, professional title, years of clinical experience, monthly night shifts, and salary satisfaction.
2. Workplace Violence: The Workplace Violence Scale (WVS) developed by Wang Peixi et al. (14) was used to assess the frequency and types of WPV experienced by nurses in the past year. The scale consists of five items covering common forms of violence: physical assault, verbal abuse, threats/intimidation, verbal sexual harassment, and physical sexual harassment. Each item is rated on a 4-point scale (0 = never, 3 = ≥4 times), with total scores ranging from 0 to 15; higher scores indicate more frequent exposure to violence. The scale demonstrated good internal consistency in this study (Cronbach’s α = 0.857).
3. Psychological Resilience: The Chinese version of the Connor-Davidson Resilience Scale (CD-RISC), revised by Xiao Nan et al. (15), was used to measure individuals’ capacity to cope with adversity. The 25-item scale includes three subdimensions: tenacity (13 items), strength (8 items), and optimism (4 items). Each item is rated on a 5-point Likert scale (0 = never, 4 = always), with total scores ranging from 0 to 100. Higher scores indicate greater resilience. In this study, the overall Cronbach’s α was 0.926, with subscale reliabilities ranging from 0.871 to 0.921.
4. Emotional Labor: The Emotional Labor Scale (ELS) revised by Luo Hong (16) was applied to assess emotional regulation strategies in professional settings. The 14-item scale comprises three dimensions: surface acting, deep acting, and natural expression. Responses are rated on a 5-point Likert scale (1 = strongly disagree, 5 = strongly agree), with higher scores indicating greater use of the respective strategy. The overall Cronbach’s α of the scale in the current study was 0.900, and the reliability of each dimension was 0.891, 0.810 and 0.815, respectively.
5. Occupational Burnout: The Maslach Burnout Inventory–Human Services Survey (MBI-HSS), revised by Feng Ying et al. (17), was used to assess burnout levels among nurses. The scale contains 22 items across three subscales: emotional exhaustion, depersonalization, and reduced personal accomplishment. Items are rated on a 7-point Likert scale (0 = never, 6 = every day), with the personal accomplishment subscale scored reversely. Total scores range from 0 to 132, with higher scores indicating more severe burnout. In this study, the overall Cronbach’s α of the scale was 0.906, and the reliability of each dimension was between 0.852 and 0.911.
6. Perceived Organizational Support: The Perceived Organizational Support Scale (POS), revised by Ling Wenquan et al. (18), was used to measure nurses’ perceived support from their organizations. The scale includes nine items rated on a 7-point Likert scale (1 = strongly disagree, 7 = strongly agree), with total scores ranging from 9 to 63. Higher scores indicate a greater sense of organizational support. The scale showed good reliability in this study (Cronbach’s α = 0.909).

### 2.5 Statistical analysis

All collected data were independently verified and double-entered into a database. Statistical analyses were conducted using SPSS version 26.0 and Mplus version 7.4.

First, Latent Profile Analysis (LPA) was performed using Mplus 7.4 based on the five items of the WVS to identify latent subgroups of WPV experiences. Starting from a one-class model, additional classes were progressively added. Model fit was evaluated using multiple indices: Akaike Information Criterion (AIC), Bayesian Information Criterion (BIC), and sample-size adjusted BIC (aBIC), with smaller values indicating better fit. Classification accuracy was assessed using entropy (range 0–1), with higher values indicating clearer class separation. Model comparisons were further validated using the Lo-Mendell-Rubin Test (LMRT) and the Bootstrap Likelihood Ratio Test (BLRT), with P < 0.05 indicating that the model fit was significantly improved compared to the previous class solution.

Second, based on the identified latent classes, further statistical analyses were conducted using SPSS 26.0. Categorical variables were described using frequencies and percentages, and group comparisons were made using Chi-square or rank-sum tests. Continuous variables were analyzed using t-tests or ANOVA, depending on distribution normality. Finally, latent profile membership was used as the dependent variable in a multivariate logistic regression model, incorporating statistically significant predictors from univariate analyses. A two-tailed P-value < 0.05 was considered statistically significant.

## 3 Results

### 3.1 Demographic characteristics of nurses

A total of 549 clinical nurses participated in this study. The majority were female (86.7%, n = 476). Age distribution was mainly between 36–45 years (32.8%), followed by 26–35 years (27.3%). Married nurses accounted for 69.2%, and unmarried nurses 20.2%. In terms of education, 76.3% held a bachelor’s degree and 12.9% had a master’s degree or higher. Regarding professional title, 46.1% were at the junior level and 43.5% at the intermediate level. Nearly half (47.4%) had over 10 years of clinical experience. Shift patterns showed that 44.8% worked 4–8 night shifts per month. Satisfaction with salary was rated “average” by 48.5% and “satisfied” by 37.5%. Contract-employed nurses comprised 72.9%, while 27.1% were on permanent positions (see **Table 1**).

**Table 1:** Demographic characteristics of nurses and univariate analysis by WPV profile (n = 549).

Variables	Type	N	High-frequency, multi-type violence group (n=152)	Low-frequency, mild violence group (n=397)	χ²/F	*p*
Gender	Male	73 (13.3)	12 (7.9)	61 (15.4)	5.321	0.021
	Female	476 (86.7)	140 (92.1)	336 (84.6)		
Age	≤25 years	92 (16.8)	35 (23.0)	57 (14.4)	9.646	0.022
	26-35 years	150 (27.3)	47 (30.9)	103 (25.9)		
	36-45 years	180 (32.8)	42 (27.6)	138 (34.8)		
	>45 years	127 (23.1)	28 (18.4)	99 (24.9)		
Marital status	Married	380 (69.2)	105 (69.1)	275 (69.3)	6.668	0.036
	Unmarried	111 (20.2)	38 (25.0)	73 (18.4)		
	Others	58 (10.6)	9 (5.9)	49 (12.3)		
Educational attainment	Junior college	59 (10.7)	9 (5.9)	50 (12.6)	9.463	0.009
	Undergraduate	419 (76.3)	115 (75.7)	304 (76.6)		
	Master degree or above	71 (12.9)	28 (18.4)	43 (10.8)		
Employment Type	Temporary contract nurse	400 (72.9)	121 (79.6)	279 (70.3)	4.837	0.028
	Tenured nurse	149 (27.1)	31 (20.4)	118 (29.7)		
Professional title	Junior professional title	253 (46.1)	73 (48.0)	180 (45.3)	11.639	0.003
	Intermediate professional title	239 (43.5)	74 (48.7)	165 (41.6)		
	Senior professional title	57 (10.4)	5 (3.3)	52 (13.1)		
Years of clinical experience	<2 years	57 (10.4)	16 (10.5)	41 (10.3)	12.422	0.006
	2–5 years	51 (9.3)	24 (15.8)	27 (6.8)		
	5–10 years	181 (33.0)	52 (34.2)	129 (32.5)		
	>10 years	260 (47.4)	60 (39.5)	200 (50.4)		
Monthly night shifts	≤1	201 (36.6)	46 (30.3)	155 (39.0)	46.707	<0.001
	2-4	246 (44.8)	50 (32.9)	196 (49.4)		
	5-8	102 (18.6)	56 (36.8)	46 (11.6)		
Salary satisfaction	≥8	77 (14.0)	47 (30.9)	30 (7.6)	55.777	<0.001
	Dissatisfied	266 (48.5)	71 (46.7)	195 (49.1)		
	Neutral	206 (37.5)	34 (22.4)	172 (43.3)		

### 3.2 Incidence of workplace violence and scale scores

The prevalence of workplace violence among nurses was 74.13%. The scores of Maslburnout Burnout Inventory, Connor-Davidson Resilience Scale, Emotional Labor Scale and Perceived Organizational Support Scale were (55.45 ± 21.31), (63.36 ± 15.24), (49.46 ± 9.26) and (42.88 ± 10.27), respectively.

### 3.3 Latent profile analysis of workplace violence

Latent profile models with 1–5 classes were fitted based on nurses’ scores on the five WPV items from the past year (see **Table 2**). As the number of classes increased, fit indices such as AIC, BIC, and aBIC declined, indicating improved model fit. With a two-class model, the entropy was 0.915, and both LMRT and BLRT were statistically significant (*P <*0.001), suggesting excellent classification accuracy and discrimination. Although a three-class model achieved perfect entropy (1.000), LMRT was non-significant (*P* > 0.05), providing no support for its superiority over the two-class solution. Four- and five-class models showed further decreases in fit indices but generated classes under 5% of the sample and non-significant LMRT tests, indicating potential overfitting and instability.

**Table 2:** Fit indices for latent profile models of workplace violence among nurses.

Class	AIC	BIC	aBIC	*P*		Enproty	Number of classes	Class probability(%)
				LMRT	BLRT			
1	6544.217	6587.298	6555.554	–	–	–	–	–
2	5460.904	5529.834	5479.043	0.0000	0.0000	0.915	152/397	27.687/72.313
3	4371.138	4465.916	4396.079	0.6190	0.6234	1.000	382/58/109	69.581/10.565/19.854
4	4131.757	4252.384	4163.500	0.1833	0.1860	0.997	23/109/35/382	4.189/19.854/6.375/69.581
5	3847.696	3994.171	3886.241	0.3100	0.3138	0.984	109/26/336/32/46	19.854/4.736/61.202/5.829/8.379

Considering model fit, interpretability, practical application, and parsimony, the two-class model was deemed optimal, and the score distributions across WPV items were plotted.

**Figure 1:**
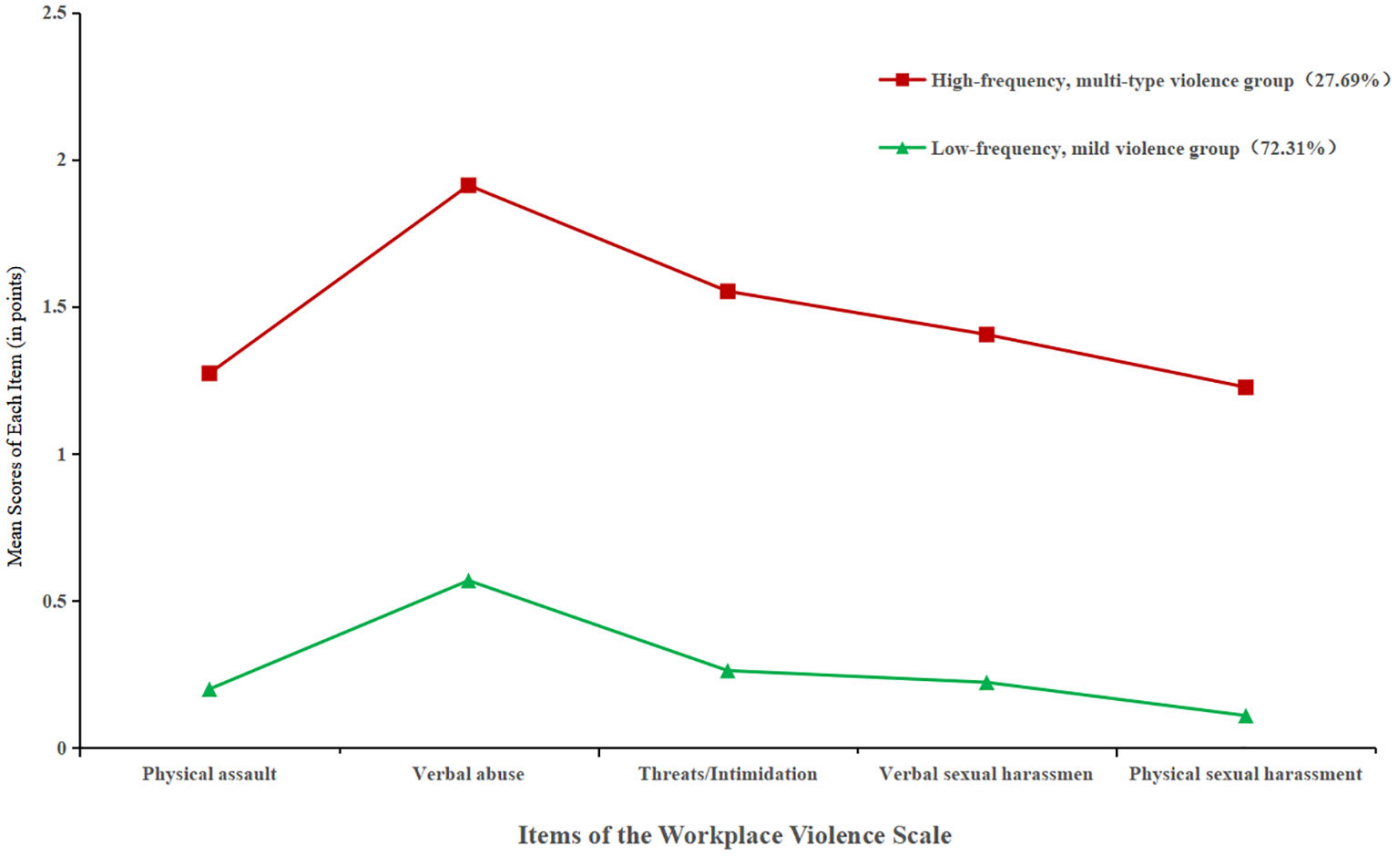
Score distributions of workplace violence items across two latent profiles of nurses.

As shown in **Figure 1**, nurses in Class 1 scored significantly higher across all violence dimensions, especially in “emotional abuse” and “threats/intimidation.” This profile, comprising 27.69% of participants, was labeled the “high-frequency, multi-type violence” group. Class 2 exhibited lower scores across dimensions and was designated the “low-frequency, mild violence” group, accounting for 72.31%.

### 3.4 Differences in demographics and psychological measures by WPV profile

Univariate analyses of demographic variables by WPV profile are presented in **Table 1**. Significant differences (*P*<0.05) were found between groups in sex, age, marital status, education level, employment type, professional title, years of clinical experience, monthly night shifts, and salary satisfaction.

Further comparison of psychological variables (**Table 3**) revealed that the “high-frequency, multi-type violence” group scored significantly higher on all dimensions of occupational burnout (emotional exhaustion, depersonalization, reduced personal accomplishment) and total burnout scores, as well as on all aspects of emotional labor (surface acting, deep acting, genuine expression) (*P*<0.01). Conversely, this group scored significantly lower on psychological resilience (resilience, self-reliance, optimism) and perceived organizational support compared to the “low-frequency, mild violence” group (*P <* 0.01).

**Table 3:** Comparison of different types of workplace violence among nurses in the scores of each scale.

Type	Score	High-frequency, multi-type violence group	Low-frequency, mild violence group	*t*	*P*
Emotional exhaustion	21.91 ± 11.09	25.90 ± 11.49	20.3 ± 10.55	5.348	0.000
Depersonalization	7.86 ± 5.97	10.26 ± 6.53	6.95 ± 5.49	5.989	0.000
Reduced personal accomplishment	25.67 ± 11.12	27.68 ± 10.75	24.90 ± 11.17	2.638	0.009
Occupational burnout	55.45 ± 21.31	63.84 ± 21.90	52.23 ± 20.21	5.884	0.000
Surface acting	24.85 ± 5.50	26.42 ± 5.22	24.25 ± 5.50	4.195	0.000
Deep acting	13.86 ± 3.24	14.64 ± 3.17	13.56 ± 3.22	3.560	0.000
Genuine expression	10.74 ± 2.49	11.34 ± 2.24	10.52 ± 2.55	3.506	0.000
Emotional labor	49.46 ± 9.26	52.41 ± 8.54	48.32 ± 9.28	4.714	0.000
Resilience	33.66 ± 9.29	29.94 ± 9.52	35.08 ± 8.81	-5.981	0.000
Self-reliance	19.66 ± 6.12	17.01 ± 5.93	20.67 ± 5.90	-6.508	0.000
Optimism	10.05 ± 3.41	9.23 ± 3.29	10.36 ± 3.41	-3.500	0.001
Psychological resilience	63.36 ± 15.24	56.18 ± 14.59	66.11 ± 14.59	-7.136	0.000
Perceived organizational support	42.88 ± 10.27	39.59 ± 11.93	44.13 ± 9.28	-4.724	0.000

### 3.5 Multivariate logistic regression analysis

Using WPV profile as the dependent variable (coded as high-frequency, multi-type violence group = 1; low-frequency, mild violence group= 0), variables that were significant in univariate analysis were entered into a binary logistic regression model (coding details in **Table 4**). Results (**Table 5**) indicated that nurses with a bachelor’s degree (OR = 3.196, *P* = 0.009) and those with a master’s degree or higher (OR = 5.364, P = 0.001) were more likely to belong to the high-frequency, multi-type violence group. Compared with nurses who were dissatisfied with their salary, those who rated it as “average” (OR = 0.311, *P* < 0.001) or “satisfied” (OR = 0.140, *P* < 0.001) had a significantly lower likelihood of being in that group. Additionally, higher resilience scores were associated with a reduced probability of high-frequency, multi-type violence (OR = 0.970, *P* = 0.001).

**Table 4:** Coding of variables used in logistic regression analysis.

Variables	Mode of assignment
Gender	0= Male (reference group), 1= Female
Age	1=≤25 years old, 2 = 26–35 years old, 3 = 36–45 years old, 4= > 45 years old
Marital status	1= Married (reference group), 2= Unmarried, 3= Divorced/deceased
Education level	Junior college =1, Undergraduate =2, Master’s degree or above =3
Employment Type	0= Temporary contract nurse (reference group), 1= Tenured nurse
Professional title	1=Junior professional title, 2= Intermediate professional title, 3= Senior professional title
Years of clinical experience	1= < 2 years, 2 = 2 years -5 years, 3 = 5 years -10 years, 4= more than 10 years
Monthly night shifts	1=≤4, 2 = 5-8, 3=≥8
Salary satisfaction	1= Dissatisfaction, 2= General, 3= Satisfied
WPV	Continuous variables
Psychological Resilience	Continuous variables
Emotional Labor	Continuous variables
Perceived Organizational Support	Continuous variables
Occupational Burnout	Continuous variables

**Table 5:** Logistic regression analysis of factors influencing wpv latent profile membership among nurses (n = 549).

Type	B	SE	Waid χ²	P	OR	95%CI
constant	0.677	1.464	0.214	0.644	–	–
Education (“Junior college and below“ as a reference)						
Undergraduate	1.162	0.443	6.866	0.009	3.196	1.340-7.620
Master’s degree or above	1.68	0.527	10.154	0.001	5.364	1.909-15.070
Salary satisfaction (with “dissatisfaction” as the reference)						
General	-1.168	0.330	12.526	<0.001	0.311	0.163-0.594
Satisfied	-1.965	0.361	29.553	<0.001	0.140	0.069-0.285
Psychological Resilience	-0.031	0.009	11.556	0.001	0.97	0.953-0.987

## 4 Discussion

### 4.1 Latent profile characteristics of workplace violence among nurses

The results of this study revealed that the overall incidence of workplace violence (WPV) among the 549 clinical nurses surveyed was 74.13%, which is lower than the 86.7% reported by Li et al. (19), but significantly higher than the global average of 44.9% for nurses (20). This indicates that nurses in China still face a relatively high risk of exposure to violence. Such discrepancies may be attributable to differences in departmental risk levels, disparities in healthcare resource allocation, variation in violence identification criteria, and the robustness of organizational support systems.

Further analysis showed that the total WPV score was 3.02 ± 3.20, suggesting a moderate overall level of violence. However, the distribution of item scores varied significantly, indicating that a subset of nurses experienced more severe forms of violence. In this context, mean scores alone fail to capture the internal heterogeneity of risk within the population, highlighting the need for structural modeling approaches to identify latent subtypes.

To address this, we employed Latent Profile Analysis (LPA), which identified two distinct patterns of WPV experiences. The “high-frequency, multi-type violence” group comprised 27.7% of the sample, with notably elevated scores across multiple dimensions such as verbal abuse, sexual harassment, and threats/intimidation. This pattern indicates cumulative and diverse exposure to violence, which may be associated with increased psychological stress and a heightened risk of occupational burnout (21). Without effective interventions and organizational support, this subgroup is more susceptible to negative professional outcomes, including emotional exhaustion, work avoidance, and even voluntary turnover—necessitating targeted identification and focused interventions (22).

In contrast, the “low-frequency, mild violence” group accounted for 72.3% of participants. Although their overall WPV scores were relatively low, this does not imply complete safety. Some nurses in this group may lack sufficient skills in risk recognition and response, making them vulnerable to unrecognized or subtle forms of violence. These individuals may hover at the edge of potential risk and thus require enhanced coping strategies, psychological resilience, and early warning system development (23, 24).

In summary, the use of LPA effectively uncovered structural heterogeneity in WPV experiences among nurses, addressing the limitations of traditional mean-based analyses in identifying high-risk subpopulations. The findings suggest that nurses with high-frequency WPV exposure should be prioritized for targeted interventions. Meanwhile, those in the low-violence group should also receive support aimed at strengthening self-protective capabilities through training, institutional safeguards, and psychological preparedness.

### 4.2 Influencing factors of latent WPV profiles among nurses

This study further explored the influencing factors associated with latent types of workplace violence (WPV) among nurses. The results indicated that educational attainment, salary satisfaction, and psychological resilience played significant roles in distinguishing between the “high-frequency, multi-type violence” and “low-frequency, mild violence” groups. These findings suggest that WPV exposure is not solely driven by external circumstances; rather, individual characteristics and internal psychological resources also exert substantial influence.

#### 4.2.1 Educational attainment and WPV

Nurses with a bachelor’s degree or higher were more likely to belong to the “high-frequency, multi-type violence” group, consistent with findings reported by Yuan et al. (25). This suggests that WPV exposure is influenced not only by external risk factors but also by deeper cognitive structures. On one hand, one of the key determinants of whether nurses report WPV is their cognitive understanding of violence (26). Nurses with higher educational levels typically receive more systematic training in professional ethics and interpersonal communication during their academic programs, leading to a clearer and more sensitive awareness of WPV definitions, types, and severity. As a result, they are more likely to classify incidents such as verbal conflicts, passive aggression, or non-verbal offenses as “violence,” resulting in higher perceived and reported frequencies (25, 27).

On the other hand, nurses with advanced education may hold higher expectations regarding their professional roles, organizational support, and patient respect. When real-world working conditions fall short of these expectations, psychological stress and emotional conflict are more likely to arise, exacerbating the perception of violence (28, 29). The findings of this study offer new evidence supporting the interaction between educational background and WPV exposure.

In WPV prevention strategies, special attention should be paid to the psychological safety and professional support of highly educated nurses. It is recommended that nursing administrators implement scenario-based training and enhance communication strategy programs targeted at this group. Moreover, establishing regular psychological counseling services and professional support platforms can help these nurses correctly identify violence, regulate emotional responses, and maintain resilience and coping abilities in high-stress and multi-role environments.

#### 4.2.2 Salary satisfaction and WPV

Salary satisfaction was identified as a key factor in differentiating the two WPV profiles. On one hand, higher income levels can enhance job satisfaction and partially alleviate the psychological burden associated with WPV (30). Previous studies have shown that salary satisfaction is closely related to emotional stability, turnover intention, and organizational commitment among nurses (31, 32). A fair salary not only meets basic financial needs but also symbolizes organizational recognition of professional contributions, thereby strengthening psychological safety and a sense of belonging while reducing emotional exhaustion and cognitive overload (33). In developing countries, income inequality is more likely to trigger dissatisfaction, heighten sensitivity to violence, and increase reporting tendencies (34).

Conversely, dissatisfaction with salary may amplify perceptions of lacking organizational support, intensifying stress responses to violent encounters (35). When nurses perceive a mismatch between their compensation and the demands, risks, and responsibilities of their job, feelings of injustice may arise. This increases the likelihood that minor conflicts or implicit offenses are interpreted as “violence.” Such cognitive magnification can reduce psychological resilience and work engagement, further aggravating negative experiences of WPV (36, 37).

Therefore, it is recommended that nursing management establish a compensation system aligned with job responsibilities and risk exposure. Incentive mechanisms should be introduced for high-risk departments and nurses with higher educational levels.Regular monitoring of salary satisfaction and WPV exposure could help identify high-risk groups and guide the timely implementation of communication training and scenario-based interventions.

#### 4.2.3 Psychological resilience and WPV

This study found that psychological resilience significantly modulates how nurses experience WPV, making it a key psychological trait in distinguishing between WPV latent classes. This is consistent with findings by Atallah et al. (38) and Chen et al. (39), further affirming that individuals’ perceptions and responses to violence are shaped not only by external situations but also by internal psychological structures.

Nurses with higher resilience levels tend to exhibit better emotional regulation and problem-solving abilities, enabling them to maintain emotional stability in the face of violence and reduce both the intensity and duration of negative experiences (38, 40). Resilience mitigates stress responses triggered by WPV and buffers its indirect effects on job satisfaction, burnout, and turnover intention (22, 41). Prior studies have shown (42) that resilient nurses are more likely to adopt active coping strategies when facing patient incivility or organizational conflicts, thereby reporting lower frequencies of perceived violence. In contrast, those with lower resilience are more prone to emotional reactivity, feelings of helplessness or victimization, and heightened sensitivity to the absence of organizational support (42, 43). When cognitive resources are insufficient to regulate emotional load, their perception of violence may become exaggerated, even interpreting boundary-level conflicts as systemic attacks. This increases psychological stress and undermines professional engagement (38, 44).

Such mechanisms are especially pronounced in high-stress, multi-role, and expectation-heavy clinical environments, particularly when individuals simultaneously lack social support and self-efficacy (4, 45).

Therefore, WPV management strategies should prioritize the assessment and development of nurses’ psychological resilience. Nursing administrators are advised to provide resilience-building programs that focus on cognitive-behavioral interventions and emotional recovery training, supported by psychological assessments and group counseling mechanisms. These measures will help nurses cultivate stable internal coping resources and improve sustainability and effectiveness in handling workplace violence.

Although occupationalburnout, perceived organizational support, and emotional labor did not reach statistical significance in the multivariate regression, they showed marked mean differences across WPV profiles. This suggests that these variables may still effect indirect or moderating roles in the psychological processing of violence, warranting further investigation in future studies.

## 5 Conclusion

This study employed Latent Profile Analysis (LPA) to identify two distinct subgroups of workplace violence (WPV) exposure among nurses and further explored the influencing factors behind these profiles. The findings revealed that educational attainment, salary satisfaction, and psychological resilience were significant determinants of WPV experience type. Specifically, nurses with higher education levels and lower salary satisfaction were more likely to be classified into the “high-frequency, multi-type violence” group, while higher psychological resilience appeared to buffer the negative effects of violence to some extent.

The study highlights the structural heterogeneity of WPV exposure patterns and emphasizes the importance of incorporating psychological characteristics into risk assessment and stratified intervention. These results provide both theoretical underpinnings and practical pathways for the precise identification of high-risk groups and the optimization of nursing management strategies.

## 6 Limitations and future directions

Several limitations should be acknowledged. First, the cross-sectional design of this study limits the ability to infer causal relationships, particularly concerning the moderating role of psychological resilience in the perception of WPV. Future research should employ longitudinal or intervention-based designs to further validate these mechanisms.

Second, the sample was primarily drawn from tertiary hospitals in Sichuan Province, China, which may limit generalizability. Future studies should include larger, multi-center samples to improve external validity.

Third, the study relied predominantly on self-reported questionnaires, which may be subject to social desirability bias. It is recommended that future research incorporate multi-source data and behavioral observation indicators to enhance the reliability and validity of measurements.

Future research should also explore the interacting mechanisms of organizational support, self-efficacy, and coping strategies in shaping WPV subtypes. In addition, empirical studies based on latent class modeling could be conducted to develop personalized intervention protocols, thereby providing a stronger evidence base for targeted psychological support strategies in clinical settings.

## Data Availability

The original contributions presented in the study are included in the article/Supplementary Material. Further inquiries can be directed to the corresponding author.
